# *IL1RL1* Gene Variants and Nasopharyngeal IL1RL-a Levels Are Associated with Severe RSV Bronchiolitis: A Multicenter Cohort Study

**DOI:** 10.1371/journal.pone.0034364

**Published:** 2012-05-04

**Authors:** Tina E. Faber, Annemieke Schuurhof, Annelies Vonk, Gerard H. Koppelman, Marije P. Hennus, Jan L. L. Kimpen, Riny Janssen, Louis J. Bont

**Affiliations:** 1 Department of Pediatrics, Medical Center Leeuwarden, Leeuwarden, The Netherlands; 2 Laboratory for Health Protection Research at the National Institute for Public Health and the Environment, Bilthoven, The Netherlands; 3 Department of Pediatric Pulmonology and Pediatric Allergology at Beatrix Children’s Hospital, University Medical Center Groningen, GRIAC Research Institute, University of Groningen, Groningen, The Netherlands; 4 Department of Pediatric Intensive Care, Wilhelmina Children’s Hospital, University Medical Center Utrecht, Utrecht, The Netherlands; 5 Department of Pediatrics, Wilhelmina Children’s Hospital, University Medical Center Utrecht, Utrecht, The Netherlands; University of Liverpool, United Kingdom

## Abstract

**Background:**

Targets for intervention are required for respiratory syncytial virus (RSV) bronchiolitis, a common disease during infancy for which no effective treatment exists. Clinical and genetic studies indicate that *IL1RL1* plays an important role in the development and exacerbations of asthma. Human *IL1RL1* encodes three isoforms, including soluble IL1RL1-a, that can influence IL33 signalling by modifying inflammatory responses to epithelial damage. We hypothesized that *IL1RL1* gene variants and soluble IL1RL1-a are associated with severe RSV bronchiolitis.

**Methodology/Principal Findings:**

We studied the association between RSV and 3 selected *IL1RL1* single-nucleotide polymorphisms rs1921622, rs11685480 or rs1420101 in 81 ventilated and 384 non-ventilated children under 1 year of age hospitalized with primary RSV bronchiolitis in comparison to 930 healthy controls. Severe RSV infection was defined by need for mechanical ventilation. Furthermore, we examined soluble IL1RL1-a concentration in nasopharyngeal aspirates from children hospitalized with primary RSV bronchiolitis. An association between SNP rs1921622 and disease severity was found at the allele and genotype level (p = 0.011 and p = 0.040, respectively). In hospitalized non-ventilated patients, RSV bronchiolitis was not associated with IL1RL1 genotypes. Median concentrations of soluble IL1RL1-a in nasopharyngeal aspirates were >20-fold higher in ventilated infants when compared to non-ventilated infants with RSV (median [and quartiles] 9,357 [936–15,528] pg/ml vs. 405 [112–1,193] pg/ml respectively; p<0.001).

**Conclusions:**

We found a genetic link between rs1921622 *IL1RL1* polymorphism and disease severity in RSV bronchiolitis. The potential biological role of IL1RL1 in the pathogenesis of severe RSV bronchiolitis was further supported by high local concentrations of IL1RL1 in children with most severe disease. We speculate that IL1RL1a modifies epithelial damage mediated inflammatory responses during RSV bronchiolitis and thus may serve as a novel target for intervention to control disease severity.

## Introduction

Respiratory syncytial virus (RSV) bronchiolitis is the most common cause of hospitalization for infants during the winter season. About 1–2% of all children are hospitalized for RSV bronchiolitis, mechanical ventilation is required in 10% of hospitalized cases [1]. Approximately half of the infants with RSV lower respiratory tract infection (LRTI) go on to have recurrent wheezing episodes until they reach school age [2]–[4]. The overall risk of concurrent bacterial infection is low, yet the reported incidence of bacterial pneumonia in children with severe RSV infection requiring ventilation ranges from 9%–44% [5]–[13]. Mechanisms underlying severe respiratory syncytial virus bronchiolitis are incompletely understood.

There is increasing evidence, from both clinical and genetic studies, that *IL1RL1* plays an important role in the development of childhood asthma [14]–[18]. *IL1RL1* gene cluster polymorphisms were found to be associated with asthma and atopy [14], [15], [18]. Human *IL1RL1* encodes the receptor for interleukin-33 (IL33) and has three isoforms, including soluble IL1RL1 (IL1RL1-a), that can modify T helper cell responses by inhibition of IL33 signalling [17], [19]–[23]. IL1RL1-a can be induced by pro-inflammatory stimuli and appears to be essential for the normal function of T helper cells [20]–[22]. Clinically, *IL1RL1* genetic polymorphisms and IL1RL1-a serum levels have been associated with severe arthritis, acute heart disease, and airway disease such as asthma [24]–[28].

## Methods

### Ethics Statement

The study protocol was approved by the institutional review board “RTPO” at the Medical Center Leeuwarden in and the medical ethics committee “METC” at the University Medical Center Utrecht in The Netherlands. All parents of hospitalized infants agreed to participate and gave written informed consent.

### Objectives

We hypothesized that *IL1RL1* plays a role in the pathogenesis of severe RSV bronchiolitis. To this end, we analyzed *IL1RL1* single-nucleotide polymorphisms (SNPs) for association with RSV disease. Furthermore, we examined the association between local IL1RL1-a concentrations and RSV disease severity.

**Table 1:** *IL1RL1* SNPs not associated with RSV bronchiolitis.

refSNP ID	RSV hospitalized infants^1^ (n = 465)					Population controls^1^ (n = 930)				Missing values	P-value^2^
**Allele**	A	G				A		G			
rs1921622	498	422				1002		826		42	0.734
rs11685480	468	454				925		915		28	0.809
rs1420101	343	571				706		1130		40	0.638
**Genotype**	AA		GA		GG		AA	GA	GG		
rs1921622	138		222		100		287	428	199	42	0.849
rs11685480	117		234		110		219	487	214	28	0.727
rs1420101	62		219		176		129	448	341	40	0.882

*IL1RL1* selected genotypes rs1921622, rs11685480 and rs1420101 are not associated with RSV bronchiolitis in hospitalized infants when compared to healthy controls in the population.

RefSNP ID is the Reference SNP (rs) Number; SNP, single-nucleotide polymorphism.

1 Number of alleles and genotypes.

2 According to χ^2^ distribution of a 2×2 table on allele or genotype frequencies.

3 Reference allele is the major allele.

**Table 2:** *IL1RL1* SNP rs1921622 associated with severe RSV bronchiolitis.

refSNP ID	RSV ventilated infants^1^(n = 81)			RSV non-ventilated infants^1^ (n = 384)			Population controls^1^(n = 930)			Missing values	P-value^2^
**Allele**	A	G		A	G		A	G			
rs1921622	73	89		425	333					10	**0.011**
rs1921622	73	89					1002	826		32	**0.017**
**Genotype**	AA	GA	GG	AA	GA	GG	AA	GA	GG		
rs1921622	17	39	25	121	183	75	287	428	199	5	**0.040**

Subgroup analysis showed an association between severe RSV disease characterized by need for mechanical ventilation and *IL1RL1* SNP rs1921622 at both the allele level, and at the genotype level (p = 0.040).

RefSNP ID is the Reference SNP (rs) Number; SNP, single-nucleotide polymorphism.

1 Number of alleles and genotypes.

2 According to χ^2^ distribution of a 2×2 table on allele or genotype frequencies.^3^

### Participants

In a multicenter cohort study, previously healthy infants under 1 year of age hospitalized with a first episode of RSV bronchiolitis were included from October 2007 until March 2009 in fifteen large urban hospitals in The Netherlands. Infants with Down syndrome, a history of wheezing, or cardiac or pulmonary pathology were excluded. RSV infection was confirmed by positive immunofluorescence in epithelial cells from nasopharyngeal aspirates (NPAs) as described previously [29], [30]. Severity of RSV illness was distinguished by need for mechanical ventilation, apparent by intubation and admission to a Pediatric Intensive Care Unit (PICU). Indication for mechanical ventilation in all centers was: severe respiratory distress or exhaustion, apnea’s, respiratory acidosis (pH<7.25), or hypoxia (oxygen saturation <92% despite oxygen therapy). For the genetic cohort study, only children of Dutch ethnicity were selected. The control population consisted of 930 healthy Dutch children that were randomly taken from the Regenboog study, a large Dutch population health examination survey [31]. In a subgroup of hospitalized patients (n = 207) with primary RSV disease, IL1RL1-a concentrations on the day of admission were measured in NPAs.

**Figure 1:**
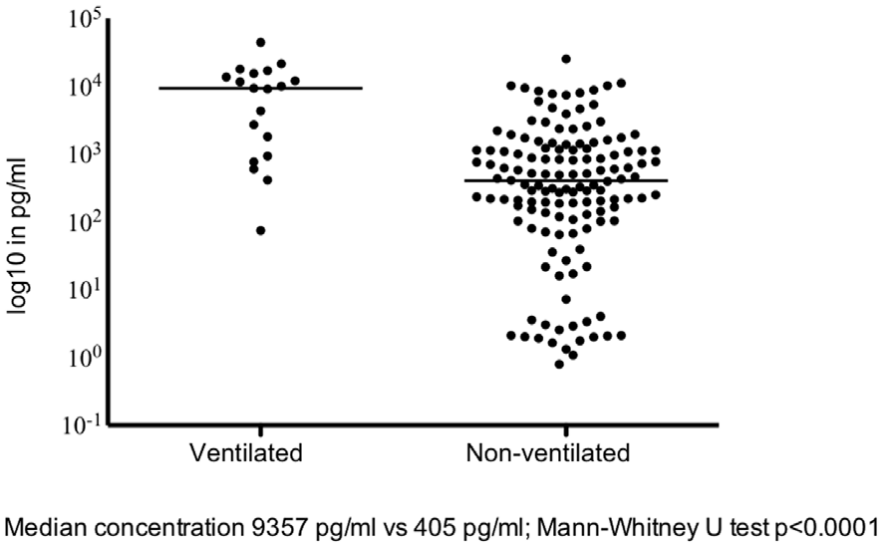
Median concentrations of IL1RL1-a in nasopharyngeal aspirates of hospitalized infants with RSV were >20-fold higher in mechanically ventilated infants at the Pediatric Intensive Care Unit (n = 19) when compared to non-ventilated infants admitted to the general pediatric ward (n = 135) (median [and quartiles] 9,357 [936–15,528] pg/ml vs 405 [112–1,193] pg/ml respectively; Mann-Whitney U test p<0.0001).

**Table 3:** Subject characteristics of infants hospitalized for RSV bronchiolitis with IL1RL1-a measured in nasopharyngeal aspirate.

	Mechanically ventilatedinfants (n = 20)	Non-mechanically ventilated infants (n = 187)	P-value
Male gender (n (%))	16 (80%)	105 (56%)	0.055 #
Gestational age (median weeks (quartiles))	38.7 (37.0–39.9)	39.7 (38.1–40.6)	**0.032** *
Born during RSV season (n (%))	17 (85%)	129 (70%)	0.197 #
Breastfeeding (n (%))	12 (60%)	111 (60%)	1.000 #
One or more older siblings (n (%)	18 (90%)	146 (78%)	0.261 #
Daycare attendance ≥1 day per week (n (%))	3 (15%)	48 (26%)	0.415 #
Atopy in the 1^st^ degree family (n (%))	11 (55%)	129 (69%)	0.216 #
Smoking during pregnancy (n (%))	6 (30%)	34 (18%)	0.233 #
Smoking in the household (n (%))	3 (15%)	15 (8%)	0.392 #
Age at admission (median days (quartiles))	70 (41–137)	51 (35–75)	0.094 *
Length of hospital stay (median days (quartiles))	14 (12–19)	4 (2–6)	**<0.001** *
Duration of ventilation (median days (quartiles))	9 (7–15)	n.a.	
IL1RL1-a measured in nasopharyngeal aspirate (n (%))	19 (95)°	135 (72)°	
Genotyping for 3 selected *IL1RL1* SNPs (n (%))	10 (50)°	120 (64)°	

# Fisher’s exact test.

* Mann-Whitney U test.

n.a. = not applicable.

Analyses were performed in nasopharyngeal aspirates of hospitalized, non-ventilated infants with respiratory syncytial virus (RSV) infection and ventilated infants at the Pediatric Intensive Care Unit with RSV.

**°**Excluded samples of poor quality had too little material to perform genotyping or an IL1RL1-a measurement.

### Investigations

Three SNPs were chosen on the basis of their potential functionality or their association with asthma in previous association studies [14], [15], [18], [32], [33]. For each SNP 1.5 µl of genomic DNA was used at 7 ng/µl. SNPs rs1921622, rs11685480 and rs1420101 present in intronic and 5′near-gene regions of the *IL1RL1* were selected for genotyping by SNP Genotyping Services at KBioscience (Hoddesdon, United Kingdom) with the KASPar technology and compared to healthy controls in the population. IL1RL1-a concentrations on the day of admission were measured in NPAs using ELISA (R&D systems, Abingdon, United Kingdom).

### Statistical Methods

For the genetic study, a convenient genetic cohort of 465 patients and 930 controls was used. Because *IL1RL1* SNPs were specifically tested, no multiple testing was required. Genotyping data were viewed graphically as a scatter plot with SNPviewer2. All SNPs were analyzed for association with RSV disease, both at the allele level (df = 1) and at the genotype level (df = 2) by Kruskal-Wallis test. Furthermore, a subanalysis was performed for association between SNPs and severe RSV disease characterized by need for mechanical ventilation. Association between IL1RL1-a concentration and RSV genotype was examined by Kruskal-Wallis test. The clinical study including 20 ventilated and 187 non-ventilated patients was designed to detect an arbitrarily chosen difference in IL1RL1-a concentrations of 180 pg/ml with a standard deviation of 200 and a power and significance of 0.8 and 0.05 respectively for a two-sided test. Mann-Whitney U test was used to compare IL1RL1-a concentrations on the day of admission in non-ventilated versus ventilated infants. All tests of significance were two-sided. P-values <0.05 were considered to be statistically significant.

## Results

In the genetic cohort study, a total of 465 Dutch children from 0–12 months of age hospitalized with primary RSV bronchiolitis were included and compared to 930 healthy Dutch controls in the population. Polymorphisms tested were in Hardy-Weinberg equilibrium. RSV bronchiolitis was not associated with selected genotypes rs1921622, rs11685480 or rs1420101 (p>0.05, table 1). Further analysis of the hospitalized infants showed an association between disease severity for the *IL1RL1* SNP rs1921622 at the allele level (p = 0.011) and the genotype level (p = 0.04) (table 2).

We further investigated our genetic finding of a potential role of *IL1RL1* in severe RSV bronchiolitis by analyzing the relationship between local IL1RL1-a concentration and disease severity. In 207 hospitalized infants with RSV bronchiolitis, 20 ventilated and 187 non-ventilated, NPA was available to measure local IL1RL1-a levels. As expected, gestational age was lower, and length of hospital stay was longer, in children requiring mechanical ventilation (38.7 weeks vs. 39.7 weeks, p = 0.032; 14 days vs. 4 days, p<0.001). All other variables, such as age at admission, male gender, breastfeeding, older siblings, smoking in the household or during pregnancy, born during RSV season, daycare attendance and atopy did not differ between groups. (table 3) Median concentrations of IL1RL1-a in nasopharyngeal aspirates were >20-fold higher in ventilated infants when compared to non-ventilated infants with RSV (median [and quartiles] 9,357 [936–15,528] pg/ml vs. 405 [112–1,193] pg/ml respectively; p<0.001, figure 1). Regression analysis showed that this effect was independent from gestational age. IL1RL1-a concentrations were not associated with any of the 3 *IL1RL1* SNPs. 3/20 (15%) of ventilated patients had evidence of concurrent bacterial pneumonia by definition of a positive bacterial culture on tracheal aspirate with single growth >100 per visual field on the day of intubation (2 patients with *Haemophilus influenzae* and 1 patient with *Moraxella catarrhalis*).

## Discussion

This study shows an association between the intron SNP rs1921622 and severe RSV disease requiring mechanical ventilation. The association between local IL1RL1-a levels and disease severity found in this study, warrants speculation on a potential role of *IL1RL1* in modifying RSV disease severity. *IL1RL1* is a member of the Toll-like receptor (TLR) superfamily and can affect Th2 responses by influencing Toll-like receptor pathway signalling. [23], [34]–[36]. *IL1RL1* is located on chromosome 2q12. *IL1RL1* translation results in 3 isoforms, of which IL1RL1-a is soluble [37]. The *IL1RL1* gene encodes the receptor for interleukine (IL) 33, and is located on mast cells, T helper type 2 (Th2) cells, regulatory T cells, and macrophages [38]–[40]. IL33 stimulates Th2 cytokine responses, such as IL4, IL5 and IL13, that induce eosinophilic influx, airway inflammation, airway hyperresponsiveness, and mucus production [23]. IL1RL1-a may act as a decoy receptor for IL33 thus affecting the inflammatory response to epithelial damage [17].

There is increasing evidence that *IL1RL1* plays an important role in the development of asthma. *IL1RL1* gene cluster polymorphisms and SNPs such as rs3771166, rs1420101 and rs1041973 have been associated with childhood asthma [14]–[18], [32]. The results of this study suggest that *IL1RL1* polymorphisms also play a role in affecting the severity of RSV disease. This is not surprising considering the common pathophysiological mechanisms of disease between asthma and RSV displayed by airway inflammation, airway hyperresponsiveness and mucus production as a result of epithelial damage.

Higher IL1RL1-a concentrations were found in children with more severe disease requiring ventilation. This is consistent with previous studies showing that higher IL1RL1-a serum concentrations were correlated with other clinically severe diseases such as acute heart disease and asthma [24], [26], [27]. IL1RL1-a in serum is also elevated in patients with acute pulmonary disease and prognostic for death within one year [28]. The results of the current study suggest that, in children with severe RSV disease, IL1RL1-a inhibits IL33 signaling and thus affects the endogenous “danger signal” that is normally stimulated by epithelial damage. The role of IL1RL1-a in RSV disease has not otherwise been studied in humans, but our findings are supported by studies in RSV sensitized mice in which the administration of anti-IL1RL1 antibodies resulted in attenuated Th2-type cytokine-associated eosinophilic airway inflammation [41].

A limitation of this part of the study is that the control group did not include a group of non-RSV infected, hospitalized patients affected by a different respiratory disease. Furthermore, there may be potential confounders, such as secondary bacterial infection. In general, rates of bacterial infection in hospitalized, febrile infants with RSV bronchiolitis is low, but rates as high as 30–40% are reported in ventilated patients [5]–[8], [42]. In the 20 ventilated infants in this study, only 3 (15%) had a positive bacterial culture with single growth >100 per visual field on the day of intubation. *Haemophilus influenzae* was found in 2 patients and *Moraxella catarrhalis* was found in 1 patient.

Local IL1RL1-a concentration in RSV disease is not associated with selected genotypes. Thus, although both RS 1921622 *IL1RL1* polymorphisms and higher local IL1RL1-a concentrations are associated with severe RSV disease, the suggestion of a gain-of-function polymorphism could not be confirmed. These results are similar to asthma studies in which associations between *IL1RL1* genotypes and acute asthma or severe asthma could be demonstrated, but not between serum IL1RL1-a and studied genotypes [15]. In contrast, one recent study showed an association between *IL1RL1* polymorphisms and serum IL1RL1-a [32]. A recent large scale genome-wide association study of asthma found associations between asthma and SNP rs3771166 on chromosome 2 implicating a role for *IL1RL1*, but also variants at different loci associated with different types of asthma, such as childhood asthma [17]. Intron 10 SNPs are in linkage disequilibrium with IL1RL1 and IL18R1. Although we attribute the effect at this locus to IL1RL1, as in previous studies [17], an IL18R1 effect cannot be excluded since SNP rs1921622 is in linkage disequilibrium with SNPs in *IL18R1*.

In conclusion, our data demonstrate for the first time a genetic association between *IL1RL1* and disease severity of RSV bronchiolitis. High IL1RL1-a production in the airways of children with severe RSV bronchiolitis suggests this molecule plays a role in modifying the inflammatory response to epithelial damage, as it does in severe asthma.
